# Mid-term clinical outcomes after net-like bridging arthroscopic rotator cuff repair: a minimum 5-year follow-up study

**DOI:** 10.1016/j.jseint.2026.101660

**Published:** 2026-02-11

**Authors:** Tomohiro Uno, Nariyuki Mura, Issei Yuki, Ryuta Oishi, Hiroshi Satake, Yuya Takakubo, Michiaki Takagi

**Affiliations:** a Department of Orthopaedic Surgery, Yamagata University Faculty of Medicine, Yamagata, Yamagata, Japan; b Department of Orthopaedic Surgery, Yoshioka Hospital, Tendo, Yamagata, Japan; c Yamagata Prefectural University of Health Sciences, Yamagata, Yamagata, Japan

**Keywords:** Arthroscopic rotator cuff repair (ARCR), Suture bridge, Net-like bridging, Radiographic assessment, Mid-term, Muscle strength, Retear rate

## Abstract

**Background:**

The purpose of arthroscopic rotator cuff repair (ARCR) is to achieve pain reduction and functional improvement for rotator cuff tears. We have adopted the net-like bridging ARCR, a double-row suture-bridge technique. The technique has demonstrated biomechanical superiority in terms of footprint coverage and contract pressure, thereby supporting clinical application. The purpose of this study is to investigate mid-term clinical outcomes and cuff integrity on magnetic resonance imaging after net-like bridging ARCR.

**Methods:**

Between 2013 and 2014, ARCR was performed on 44 patients with symptomatic full-thickness rotator cuff tears, specifically focusing on those with a maximum tear size of 20 mm or more. Of these patients, 22 patients who consented to participate and returned for follow-up evaluation at a minimum of five years post-operatively were included. The mean age at surgery was 65.8 years (range, 54.6-79.7 years). Active range of motion, clinical score, Cofield classification, and Sugaya classification were investigated. Retear was defined according to the Sugaya classification as types 4 and 5. A *P* value less than .05 was significant.

**Results:**

Mean follow-up period was 71.3 months (range, 62.9-79.6 months). There were 20 males (91%) and 2 females. The Cofield classification was as follows: medium (6 cases), large (8 cases), and massive (8 cases). For active range of motion, flexion increased from pre-operative 131° ± 42° to final 159° ± 12° (*P* < .05). The Japanese Orthopedic Association score also improved from a pre-operative value of 62.1 ± 11.5 points to 93.9 ± 5.1 points at final follow-up (*P* < .01). The Constant score at final follow-up was 88.2 ± 11.9 points. Sugaya classification at 1 year and final follow-up are shown below, there was no significant difference: 1 year, type 1: 10; type 2: 3; type 3: 3; type 4: 5; type 5: 1; at final follow-up, type 1: 9; type 2: 3; type 3: 4; type 4: 2; type 5: 4. The retear rate was 27.3%, and no new retears occurred between 1 year post-operatively and the final follow-up.

**Conclusion:**

Net-like bridging ARCR for medium-sized or larger full-thickness rotator cuff tears (≥20 mm) results in a retear rate of 27.3%, with no new retears beyond 1 year at a mean follow-up of 71.3 months (minimum 5 years), and sustained muscle strength in patients without retear. Despite the low follow-up rate, two-time-point evaluation in large tears (≥20 mm) provides clinically relevant mid-term outcomes.

Arthroscopic rotator cuff repair (ARCR) is widely performed to alleviate pain and improve shoulder function. The primary purpose of ARCR is to achieve pain reduction and functional improvement. Retear after ARCR is the cause of poor post-operative results.^4^^,^^14^^,^^22^^,^^24^^,^^34^ To enhance tendon-to-bone healing, various fixation strategies have been developed, including single-row (SR), double-row (DR), and DR suture bridge (SB) techniques. The superiority of SR, DR, and SB fixation has been discussed in several comparative studies, primarily reporting short-term outcomes with a minimum follow-up of 1 year.^16^ The mid- to long-term retear rate is reported to be 47%-92% with the SR method and 23% with the DR method.^1^^,^^17^^,^^21^^,^^30^^,^^31^ As the most recent development among rotator cuff repair techniques, the SB construct provides improved tendon-to-bone contact and compression over the anatomic footprint, thereby potentially enhancing healing.^16^ However, in a meta-analysis by Hein et al,^16^ even with the SB technique, the retear rate after a follow-up period of more than 12 months remained high, with rates of 28% for large and 40% for massive tears. Structural failures still occur with SB repairs, and retear rates range from 11% to 94%.^11^^,^^14^^,^^17^^,^^19^^,^^24^^,^^26^

The SB technique has been biomechanically validated for its superior footprint coverage and initial fixation strength in ARCR. However, the clinical benefit of medial-row knot tying remains a matter of discussion. While biomechanical analyses favor knotted medial row for improved contact pressure and construct stability, several clinical studies have reported comparable retear and functional outcomes between knotted and knotless medial-row constructs during short- to mid-term follow-up periods.^13^^,^^20^^,^^39^^,^^40^ Takeuchi et al^7^ suggest that knotless techniques may be associated with a lower incidence of medial cuff failure (type 2 retear), while knot tying may have a higher rate of this complication. However, overall retear rates remain comparable, and type 2 failures may be associated with worse function.^35^ Knotless DR transosseous-equivalent rotator cuff repair techniques show a downward trend in retear rate.^12^ Sano et al^32^ reported that knotted transosseous-equivalent repair induces greater stress concentration at the medial row than knotless repair, and excessive medial-knot tension may be a contributing factor to medial retear. We have adopted the net-like bridging ARCR, one of the SB techniques, to reduce stress concentration in the medial row, which is associated with a lower retear rate.^37^ There are no reports of mid-term results after net-like bridging ARCR. Whether the retear rate increases over the long-term following rotator cuff repair remains uncertain. The purpose of this study was to describe the mid-term clinical and structural outcomes of ARCR using a net-like bridging technique. We hypothesized that this technique would demonstrate acceptable clinical outcomes at mid-term follow-up. We hypothesized that this technique would demonstrate acceptable clinical outcomes at mid-term follow-up.

## Materials and methods

### Study design and population

This study was a single-arm retrospective study conducted in accordance with the World Medical Association Declaration of Helsinki and approved by the institutional ethics committee in Yoshioka Hospital, and the study number is YHTIB-2019-007. Between 2013 and 2014, ARCR was performed on 53 patients with symptomatic full-thickness rotator cuff tears. Of these, 5 patients who underwent other repair techniques, 2 patients who underwent partial repair, and 2 patients with isolated subscapularis tendon tears were excluded, resulting in a final cohort of 44 patients with a maximum tear size of 20 mm or more. Of these patients, 22 patients who consented to participate and returned for follow-up evaluation at a minimum of five years post-operatively were included in this study. Their data were retrospectively reviewed. The mean age at surgery was 65.4 years (range, 54.6-79.7 years). The demographic data, active range of motion (flexion, abduction, external rotation at side, internal rotation), isometric shoulder strength of the abductor at 45° and the external rotator at the side using MICROFET (Hoggan Health Industries Inc., USA), Japanese Orthopedic Association (JOA) score,^18^ Constant score,^8^ and DeOrio and Cofield classification^10^ were investigated. Magnetic resonance imaging (MRI) examinations were performed in all 22 patients at both 1 year post-operatively and at the final follow-up, and cuff integrity was assessed using Sugaya classification by the same shoulder specialist.^33^ Retear was defined according to the Sugaya classification as types 4 and 5. To measure range of motion (ROM), abduction was measured from the back in degrees between the upper extremity and the thorax. External rotation was measured in degrees between the arm and thorax while keeping the forearm in adduction and fixing the elbow at 90°. Internal rotation ROM was measured as the vertebral level the patient could reach with the tip of the thumb. Vertebral levels were equated with sequential numbers for statistical analysis (1-12 for T1-T12, 13-17 for L1-L5, and 18 for the sacrum or buttock).^6^ The isometric strength was measured 3 times, and the average was used as the final value. External rotation was measured with arm at side.

### Surgical procedure

All the surgical procedures were performed by one senior shoulder surgeon who was a member of the Japanese Board of Orthopedic Surgery. Under general anesthesia the patients were placed in a beach-chair position, and net-like bridging ARCR was performed.^29^

**Figure 1:**
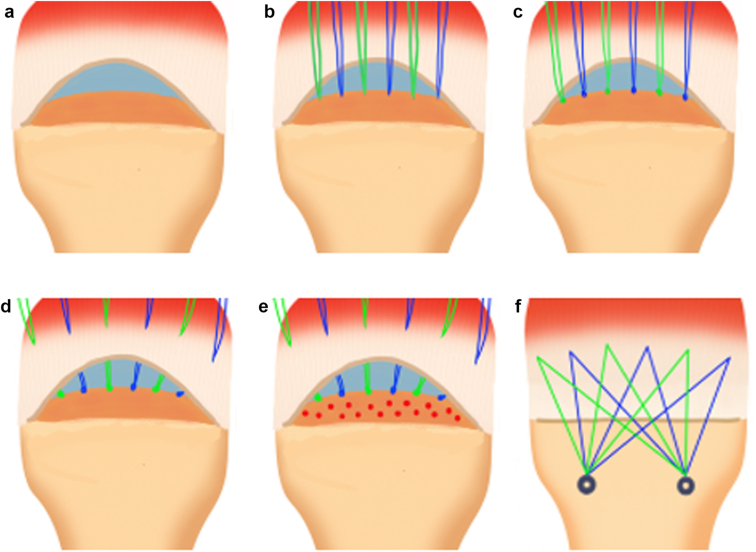
Schematic diagram illustrating the net-like bridging ARCR. (**a**) Footprint preparation using shaver and radiofrequency device. (**b**) Placement of all-suture anchors in the medial footprint. (**c**) Creation of stopper knots with all sutures to prevent slippage. (**d**) Sequential suture passage across the tear with 5-7 mm spacing. (**e**) Creation of bone marrow vents in the footprint. (**f**) Completed net-like bridging repair construct. *ARCR*, Arthroscopic rotator cuff repair.

First, an arthroscope was introduced to the glenohumeral joint through a posterior viewing portal, and the glenohumeral joint was examined. After the intra-articular procedures, the arthroscope was moved to the subacromial space. Excessive bursal tissue was débrided for better visualization. Acromioplasty was performed using a motorized burr in all the cases. Then, the supraspinatus and infraspinatus were repaired. Figure 1 shows a schematic diagram of the net-like bridging ARCR. After preparing the footprint using a shaver and a radiofrequency device (Fig. 1*a*), all-suture anchors were placed in a medial footprint (Fig. 1*b*), and stopper knots were created with all sutures (Fig. 1*c*). A stopper knot, in this context, is a square knot formed to secure the sutures and prevent slippage within the anchor. Beginning posteriorly, sutures are then evenly passed using a suture-passing device across the entire breadth of the tear, with both ends of each suture retrieved so that 2 suture limbs are obtained (Fig. 1*d*). Each pass is spaced about 5-7 mm. And then, the bone marrow vent was created (Fig. 1*e*). The bone is exposed just lateral to the greater tuberosity using a radiofrequency ablator in preparation for lateral-row anchor placement, and 2 lateral-row anchors are used. The anterior lateral-row anchor is placed lateral to the anterior margin of the rotator cuff tear, and the posterior anchor is positioned lateral to the almost posterior margin. Alternating sutures are retrieved from the lateral portal and passed through the lateral-row anchor. An awl was used to create the socket for the anterior anchor, which is located just lateral to the footprint in the greater tuberosity, with the arm in slight abduction and external rotation. The anterior anchor was placed into the socket, and the sutures were tensioned. The lateral anchor is then fully seated, and the sutures were cut flush with the anchor (Fig. 1*f*). This process was then repeated with the remaining sutures and a second lateral-row anchor, with the arm in slight external rotation. Sutures from the medial-row all-suture anchor passed through a single point, and each end is secured laterally by anchors on the greater tuberosity in a net-like pattern, a technique referred to as the net-like bridging ARCR. Finally, a 3-mm drain was placed in the subacromial space.

### Post-operative regimen

The affected arm was immobilized with an abduction brace immediately after surgery. ROM exercises of the elbow and distal on the day after surgery. Passive shoulder ROM exercises were initiated at 1 week post-operatively for medium tears and at 3 weeks post-operatively for large to massive tears, with the shoulder protected in an abduction brace. The immobilization period was 4 weeks for medium tears and 6 weeks for large and massive tears. The patients were instructed to perform passive assisted stretching exercises for a mean period of 3 weeks. Active elevation was gradually allowed from around 12 weeks in all the cases.

### Statistical analyses

Values are presented as mean and standard deviation. Statistical analysis was conducted using unpaired *t*-test and repeated-measures analysis of variance followed by Bonferroni post hoc multiple comparisons test. Fisher exact test was also used where appropriate. All statistical analyses were performed using GraphPad Prism 10 (GraphPad Software). Multiple comparisons were performed with Bonferroni correction applied to control for type I error. In addition, a post hoc power analysis using G∗Power (version 3.1.9.6) achieved a statistical power of 0.61 (two-tailed, effect size dz = 0.5, α = 0.05, total sample size = 22). 95% confidence intervals (CIs) were calculated. A *P* value < .05 was considered statistically significant.

## Results

**Figure 2:**
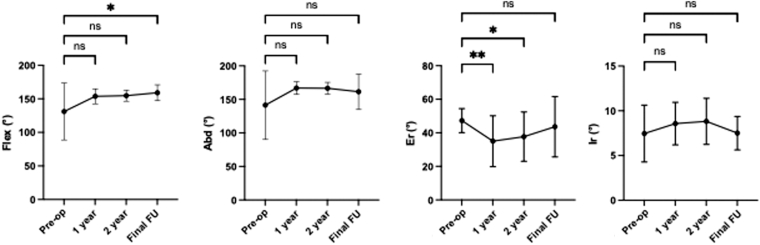
Comparison of pre-operative and post-operative active range of motion. The pre-op and post-operative [1 year, 2 year, and final FU] aROM. These figures show the aROM for Flex, Abd, Er, and Ir. Data are presented as mean ± standard deviation. Statistical analysis was performed using repeated-measures ANOVA followed by Bonferroni post hoc multiple comparisons test (∗*P* < .05, ∗∗*P* < .01). *FU*, follow-up; *aROM*, active range of motion; *Flex*; flexion; *Abd*, abduction; *Er*, external rotation; *Ir*, internal rotation; *pre-op*, pre-operative; *ANOVA*, analysis of variance; *ns*, not significant.

There were 20 males (91%) and 2 females. The mean follow-up period was 71.3 months (range, 62.9-79.6 months). The mean age at surgery was 65.8 years (range, 54.6-79.7 years). No significant complications related to ARCR were found. The mean pre-operative JOA score was 62.1 ± 11.5 points. The DeOrio and Cofield classification was medium: 6 cases, large: 8 cases, and massive: 8 cases. For active flexion, the pre-operative mean was 131° ± 42° (Fig. 2). Flexion increased to 154° ± 11° at 1 year (*P* = .13, 95% CI: –41.6 to 4.0) and to 155° ± 8° at 2 years (*P* = .07, 95% CI: –48.5 to 1.2). By the final follow-up, flexion had improved significantly to 159° ± 12° (*P* = .03, 95% CI: –53.1 to −2.8). For active abduction, there were no significant differences between the pre-operative value (141° ± 51°) and those at 1 year (167° ± 10°, *P* = .20, 95% CI: −49.0 to 7.1), 2 years (167° ± 9°, *P* = .09, 95% CI: −53.1 to 2.7), or the final follow-up (161° ± 26°, *P* = .42, 95% CI: −53.9 to 13.9). For active external rotation, the pre-operative mean was 47° ± 7°. External rotation decreased significantly at 1 year (35° ± 15°, *P* = .007, 95% CI: 3.0-21.3) and at 2 years (38° ± 15°, *P* = .02, 95% CI: 1.2-17.9). However, by the final follow-up (44° ± 18°, *P* > .99, 95% CI: −6.0 to 13.3), the value was not significantly different from the pre-operative level. For internal rotation, there were no significant differences between the pre-operative value (7.5 ± 3.2) and those at 1 year (8.6 ± 2.4, *P* = .15, 95% CI: −2.7 to 0.3), 2 years (8.8 ± 2.6, *P* = .16, 95% CI: −3.1 to 0.4), or the final follow-up (7.5 ± 1.9, *P* > .99, 95% CI: −1.7 to 1.6).

**Figure 3:**
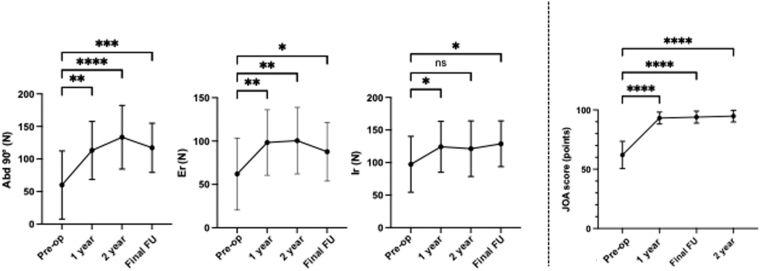
Comparison of pre-operative and post-operative isometric strength and clinical scores. The pre-op and post-operative [1 year, 2 year, and final FU] isometric shoulder strength of the abductor at 90°, the external and internal rotator at the side using MICROFET, and JOA score. Data are presented as mean ± standard deviation. Statistical analysis was performed using repeated-measures ANOVA followed by Bonferroni post hoc multiple comparisons test (∗*P* < .05, ∗∗*P* < .01, ∗∗∗*P* < .001, ∗∗∗∗*P* < .0001). *FU*, follow-up; pre-op; pre-operative; *ANOVA*, analysis of variance; *JOA*, Japanese Orthopedic Association; *ns*, not significant.

Abduction strength at 90° increased significantly from a pre-operative value of 60.1 ± 52.4 N to 113.2 ± 44.6 N at 1 year (*P* = .001, 95% CI: −81.4 to −19.2, Fig. 3), 133.4 ± 48.9 N at 2 years (*P* < .0001, 95% CI: −106.0 to −39.0), and 117.2 ± 37.8 at the final follow-up (*P* = .0002, 95% CI: −88.8 to −27.4). External rotation strength also improved significantly, increasing from a pre-operative value of 62.0 ± 41.5 N to 98.3 ± 37.8 N at 1 year (*P* = .002, 95% CI: −56.6 to −12.0), 100.5 ± 38.4 N at 2 years (*P* = .002, 95% CI: −61.0 to −13.0), and 87.7 ± 33.9 N at final follow-up (*P* = .02, 95% CI: −47.6 to −3.7). Similarly, internal rotation strength significantly increased from pre-operative 97.3 ± 42.9 N to 124.2 ± 39.2 N at 1 year (*P* = .01, 95% CI: −52.9 to −5.5). Although the improvement at 2 years (121.3 ± 42.7 N, *P* = .11, 95% CI: −47.9 to 3.6) was not statistically significant, internal rotation strength showed a significant increase at the final follow-up, reaching 128.8 ± 35.1 N (*P* = .02, 95% CI: −58.4 to −4.6).

The JOA score also improved from a pre-operative value of 62.1 ± 11.5 points to 93.1 ± 5.0 points at 1 year (*P* < .0001, 95% CI: −38.2 to −23.1) and 94.8 ± 4.9 points at 2 years (*P* < .0001, 95% CI: −40.9 to −26.0), and was 93.9 ± 5.1 points at final follow-up (*P* < .0001, 95% CI: −39.1 to −24.6, Fig. 3). The Constant score at final follow-up was 88.2 ± 11.9 points.

**Figure 4:**
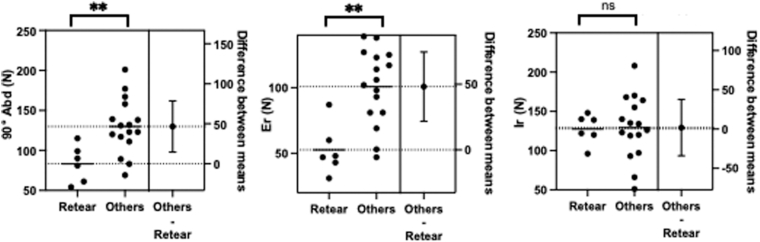
Comparison of isometric shoulder strength at final follow-up between the retear group and the no-retear group. The 6 cases in the retear group demonstrated significantly lower muscle strength compared to the no-retear group, with abduction strength at 90° (90°Abd) measuring 46.5 ± 15.3 N and Er strength measuring 48.2 ± 12.7 N. There was no significant difference in Ir strength. Unpaired *t*-test: ∗∗*P* < .01. *Abd*, abduction; *Er*, external rotation; *Ir*, internal rotation; *ns*, not significant.

Sugaya classification at 1 year after surgery and final follow-up are shown below (Table I). The retear rate was 27.3%, and there was no significant difference (*P* = .55); 1-year results, type 1: 10, type 2: 3, type 3: 3, type 4: 5, type 5: 1. Final follow-up results, type 1: 9, type 2: 3, type 3: 4, type 4: 2, type 5: 4. Although the retear rate did not differ between 1-year and final follow-up, 3 cases classified as Sugaya type 4 at 1 year progressed to Sugaya type 5 at the final follow-up. The retear rate was zero of 6 cases for medium-sized tears and 6 of 16 cases for large tears. In the 6 cases with retear, at the final follow-up, the retear group demonstrated significantly lower muscle strength compared to the no retear group, with abduction strength at 90° measuring 46.5 ± 15.3 N and external rotation strength measuring 48.2 ± 12.7 N (Fig. 4, *P* < .01).

**Table I:** Post-operative radiographic findings using the Sugaya classification.

Follow-up	Sugaya classification				
	1	2	3	4	5
Short-term 12.0 mo	10	3	3	5	1
Mid-term 71.3 mo (63-80)	9	3	4	2	4

Radiographic findings using the Sugaya classification at short-term (12.0 mo) and mid-term (mean, 71.3 mo) follow-up. The distribution of types remained relatively stable, with no significant differences between the 2 time points. Fisher exact test: *P* = .55.

## Discussion

This study evaluated the mid-term clinical outcomes of net-like bridging ARCR, one of the DR SB techniques, for symptomatic full-thickness rotator cuff tears classified as medium size or larger. In this study, the retear rate following the net-like bridging for symptomatic full-thickness rotator cuff tears with a maximum tear size of 20 mm or more was 27.3% at a mean follow-up of 71.3 months, and no new retears were observed beyond 1 year post-operatively. These findings suggest that retears tend to occur within the first post-operative year, with no additional retears observed during the subsequent mid-term period of at least five years.

Nine point one percent to 30% of retears occur within 3 months post-operatively.^2^^,^^3^ The area of the rotator cuff tendon becomes ischemic, and rotator cuff healing occurs by the formation of granulation tissue at the tendon–bone interface, possibly leading to retearing.^23^^,^^36^ Adequate distribution of compression forces across the footprint is essential and the net-like bridging ARCR technique, which uses multiple sutures to disperse stress concentration, may improve this issue.

There are a few reports to assess the retear with MRI findings. According to the systematic review of over 10 years of follow-up after ARCR, the retear rate on MRI is 49.2%.^9^ Galatz et al^14^ demonstrated that most structural failures occurred within 6-12 months post-operatively, and recurrent tears were observed in 17 of the 18 patients (94.4%). Buyukdogan et al reported an overall retear rate of 21.3% at a mean follow-up of 10.9 years after ARCR. According to Elliott et al,^5^ a recurrent tear was observed in 37% of patients at a mean follow-up of 16.25 years after surgery. In this study, the retear rate at 1 year post-operatively was 27.3%, and it remained unchanged at the final follow-up of 71.3 months, with no additional retears observed beyond 1 year. Taken together, the retear rate observed in the present study is comparable to, or slightly lower than, those reported in previous long-term studies. Given that our cohort comprised patients with relatively large rotator cuff tears ≥20 mm, these findings support the effectiveness of net-like bridging ARCR in achieving durable structural integrity in this larger tear population. Although MRI has been widely used in clinical studies, no previous research has assessed changes in MRI findings over multiple time points. Acromioplasty was routinely performed in all our cohort, and recent high-quality randomized controlled trials and systematic reviews have shown that adding acromioplasty to ARCR is associated with a significantly lower reoperation rate.^38^^,^^42^ We cannot exclude the possibility that routine acromioplasty may have influenced the retear rate or contributed to the absence of an increase in retear rates over the long term.

Retear after ARCR is one of the main causes of poor post-operative outcomes.^4^^,^^14^^,^^22^^,^^24^^,^^34^ Compared to intact repair, retears have been reported to result in significantly reduced flexion and abduction ROM, as well as decreased strength at 90°of abduction, at 1-year follow-up after ARCR using the SB procedure.^11^ In this study, shoulder flexion gradually improved over time and was significantly better at the final follow-up compared with the pre-operative value. In contrast, external rotation was significantly decreased at 1 and 2 years post-operatively compared with the pre-operative level; however, it recovered to a level comparable to baseline by the final follow-up, with no significant difference from the pre-operative value. This transient limitation in external rotation may be attributable to post-operative stiffness, capsular tightening, or tension related to the repair construct. The subsequent recovery suggests that this restriction is largely reversible with appropriate rehabilitation and long-term follow-up. To the best of our knowledge, no studies have reported the minimal clinically important difference for shoulder ROM or muscle strength following rotator cuff repair. Significant improvement of ROM in forward flexion and external rotation corresponded to an increase of more than 20% from the pre-operative value. Similarly, significant improvements in muscle strength were associated with an increase of over 80% in abduction strength and over 20% in internal rotation strength. There were no significant differences in abduction and internal rotation ROM compared to pre-operative values during the follow-up period. Pre-operative ROM has been identified as a strong predictor of post-operative shoulder mobility following rotator cuff repair.^28^ This emphasizes the importance of a comprehensive approach that includes pre-operative rehabilitation to enhance shoulder mobility, intraoperative interventions to minimize stiffness, and early post-operative rehabilitation to optimize functional recovery.

During the post-operative follow-up period, we observed an average marked improvement of more than 30 points, reflecting both pain relief and functional recovery. The improvement in the JOA score exceeded the established minimal clinically important difference of 19.5 points in ARCR, and most patients achieved the patient acceptable symptom state threshold, indicating a satisfactory post-operative state.^43^ Marrero et al reported that 76% of shoulders showed grade 5 abduction strength and 94% achieved forward flexion >150° at over 10 years follow-up after SR repair. However, that study included tears of all sizes and did not evaluate the retear rate.^25^ In this study, patients with retears exhibited significantly lower muscle strength in abduction and external rotation compared to those without retears, highlighting the functional impact of structural failure and underscoring the importance of secure tendon healing. The improvement in muscle strength across all directions suggests that surgical treatment contributes to the improvement of muscle strength, and this effect is maintained over mid- to long-term period. Clinical outcomes have been reported to remain stable in studies with a minimum follow-up of five years.^15^ The sustained improvement in JOA scores and muscle strength from 1 year post-operatively to the final follow-up suggests a durable functional outcome following the net-like bridging ARCR.

This study has several limitations, including a small sample size and its retrospective design. This study did not include a comparison group. In addition, the very low proportion of female patients may further limit the generalizability of the results. Conducting long-term prospective follow-up studies is inherently challenging. Therefore, future studies with larger cohorts and comparative designs are warranted to validate the effectiveness of this technique and to clarify prognostic factors influencing retear risk and functional recovery. Second, the number of patients who underwent ARCR in this study was small. Because small tears have a high healing rate, the study focused on full-thickness rotator cuff tears of medium size or larger, which resulted in a limited sample size. Because the study included only patients who consented to follow-up more than five years after surgery, the follow-up rate was reduced to 50%, which constituted a serious limitation and markedly increased the risk of selection and attrition bias. This high loss to follow-up has led to an underestimation or overestimation of the true retear rate, which limits the generalizability of our findings. Another limitation of this study is the marked sex imbalance, with males accounting for 91% of the study population. Although previous studies have reported that rotator cuff tears tend to be more prevalent in men,^27^^,^^41^ this pronounced male predominance may limit the generalizability of our findings, particularly to female patients. Although multiple ROM parameters were included in the analysis, internal rotation was assessed using vertebral level–based numerical conversion. The lack of validation of intra- and interobserver reliability for this method is an additional limitation.

Another limitation of this study is the potential influence of the learning curve, as all procedures were performed by a single surgeon. Surgical technique and outcomes may have evolved over time with increasing experience, which could have affected the clinical results. In addition, although multiple ROM parameters were included in the analysis, internal rotation was assessed using vertebral level–based numerical conversion. The lack of validation of intra- and interobserver reliability for this method should be considered an additional limitation.

## Conclusion

This study elucidates the mid-term clinical outcomes of net-like bridging ARCR for full-thickness rotator cuff tears of medium size or larger, demonstrating a retear rate of 27.3% with no additional failures occurring beyond 1 year, over a mean follow-up of 71.3 months (minimum 5 years). Muscle strength improvements in all directions were sustained mid- to long-term when no retear occurred.
